# Hypocalcemia in Dairy Cows: A Systematic Review of Metabolic Implications and Management Strategies

**DOI:** 10.3390/life16071082

**Published:** 2026-06-28

**Authors:** Elena Stancheva, Toncho Penev

**Affiliations:** Department of Ecology and Animal Hygiene, Faculty of Agriculture, Trakia University, 6000 Stara Zagora, Bulgaria; elena.stancheva@trakia-uni.bg

**Keywords:** hypocalcemia, dairy cows, subclinical hypocalcemia, calcium homeostasis, calcium supplementation, ionized calcium, transition period

## Abstract

**Background/Objectives:** Hypocalcemia is a major transition-cow disorder in dairy cattle, with clinical and subclinical forms differing in detectability, severity, timing, and herd-level consequences. This systematic review integrates evidence on calcium (Ca) homeostasis, classification of clinical hypocalcemia (CHC) and subclinical hypocalcemia (SCH), diagnostic interpretation, risk factors, systemic effects, and preventive and therapeutic strategies in dairy cows. **Methods:** Following the Preferred Reporting Items for Systematic Reviews and Meta-Analyses (PRISMA) 2020 statement, PubMed, ScienceDirect, SpringerLink, and CAB Abstracts were searched in February 2026 for 1994–2025 publications, and 23 reports were included in a qualitative synthesis; meta-analysis was not performed because of methodological and outcome heterogeneity. **Results:** The evidence indicates that hypocalcemia should be interpreted as a failure of homeorhetic adaptation to abrupt mammary Ca export rather than as a simple mineral deficiency. The parathyroid hormone–vitamin D axis, skeletal Ca mobilization, renal Ca conservation, intestinal Ca absorption, magnesium (Mg) status, dietary cation–anion difference (DCAD), dry matter intake, parity, and acid–base balance jointly determine whether blood Ca is maintained during early lactation. Total calcium (tCa) thresholds are useful decision aids for herd-level monitoring, but their interpretation depends on sampling time, parity, persistence pattern, clinical signs, and the relationship between tCa and ionized calcium (iCa). Subclinical hypocalcemia is most relevant when it is delayed, persistent, or occurs in high-risk cows because reduced Ca availability can impair smooth muscle function, feed intake, immune competence, uterine health, and metabolic resilience. Management should therefore combine prepartum ration control, Mg adequacy, DCAD and urine pH monitoring, selective Ca testing in high-risk cows, targeted oral Ca supplementation for standing cows, and intravenous Ca treatment for recumbent CHC cases. **Conclusions:** The evidence supports a risk-based, context-aware strategy rather than universal threshold-driven treatment.

## 1. Introduction

Calcium (Ca) is an essential macromineral in dairy cows that is involved in neuromuscular excitability, gastrointestinal motility, endocrine signaling, and immune function. Extracellular Ca is required for excitation–contraction coupling of smooth muscle and for maintaining gastrointestinal function during the periparturient period [1,2]. Disturbances in Ca homeostasis impair immune function, particularly neutrophil activity, through intracellular Ca-dependent mechanisms, thereby increasing susceptibility to postpartum infections and metabolic disorders [2]. During the transition period (approximately three weeks prepartum to three weeks postpartum), metabolic demands increase due to the onset of lactation and fetal development. When regulatory mechanisms are insufficient, hypocalcemia, including both clinical and subclinical forms, develops and represents one of the most common metabolic disorders in early lactation [1]. Clinical hypocalcemia (CHC), also known as milk fever, typically occurs within 48–72 h after calving and is characterized by reduced serum Ca concentrations (≤1.38 mmol/L) and neuromuscular dysfunction. It is associated with decreased milk production and an increased risk of retained placenta, metritis, mastitis, and abomasal displacement [2,3]. Subclinical hypocalcemia (SCH), defined as reduced Ca concentrations without apparent clinical signs (≤2.0 mmol/L) during the first 24–72 h postpartum, is more prevalent and negatively affects immune function, feed intake, and reproductive performance [4].

Subclinical hypocalcemia may persist beyond the immediate postpartum period and recur during lactation, particularly in multiparous and high-producing cows [4]. Effective monitoring requires assessment of both total calcium (tCa) and ionized calcium (iCa), as well as identification of high-risk groups. Preventive and therapeutic strategies are widely applied; however, variation in protocols, timing, and herd management leads to inconsistent outcomes [1,2]. Therefore, a structured evaluation of the available evidence is necessary to determine the effectiveness and limitations of these approaches.

The aim of this systematic review is to assess current evidence on monitoring, prevention, and management strategies for hypocalcemia in dairy cows, and to identify inconsistencies and knowledge gaps.

### Purpose and Objectives

The objectives of this study are to:

(1) Evaluate the evidence on the physiology, classification, and clinical manifestations of hypocalcemia in dairy cows.
(2) Assess monitoring methods, including biochemical and clinical indicators.
(3) Compare preventive and therapeutic strategies, including nutritional and pharmacological interventions.
(4) Identify limitations and inconsistencies in existing studies and protocols.
(5) Identify knowledge gaps and priorities for future research.

## 2. Materials and Methods

This systematic review was conducted in accordance with the Preferred Reporting Items for Systematic Reviews and Meta-Analyses (PRISMA) 2020 guidelines. The protocol was not registered. The review question, eligibility criteria, screening procedure, extraction fields, methodological quality domains, and qualitative synthesis approach were defined before final data extraction and are reported transparently below.

### 2.1. Data Sources and Search Strategy

A systematic literature search was performed in PubMed (National Center for Biotechnology Information, NCBI), ScienceDirect (Elsevier), SpringerLink, and CAB Abstracts. Supplementary searching was performed in Google Scholar and by checking reference lists of eligible articles and relevant veterinary guidance sources. Google Scholar and reference list screening were used only to identify potentially missed eligible records and were not treated as independently reproducible database counts.

The database-specific search strings were adapted to each platform while preserving the same concept blocks.

PubMed was searched using: ((“dairy cow*”[Title/Abstract] OR cattle[Title/Abstract] OR bovine[Title/Abstract] OR Holstein[Title/Abstract] OR Jersey[Title/Abstract]) AND (hypocalcemia[Title/Abstract] OR hypocalcaemia[Title/Abstract] OR “milk fever”[Title/Abstract] OR “subclinical hypocalcemia”[Title/Abstract] OR “subclinical hypocalcaemia”[Title/Abstract]) AND (“calcium metabolism”[Title/Abstract] OR “ionized calcium”[Title/Abstract] OR “total calcium”[Title/Abstract] OR monitoring[Title/Abstract] OR diagnosis[Title/Abstract] OR prevention[Title/Abstract] OR treatment[Title/Abstract] OR “oral calcium”[Title/Abstract] OR “calcium bolus”[Title/Abstract] OR “dietary cation-anion difference”[Title/Abstract] OR DCAD[Title/Abstract])).

ScienceDirect and SpringerLink were searched using: (“dairy cow” OR “dairy cows” OR cattle OR bovine OR Holstein OR Jersey) AND (hypocalcemia OR hypocalcaemia OR “milk fever” OR “subclinical hypocalcemia” OR “subclinical hypocalcaemia”) AND (“calcium metabolism” OR “ionized calcium” OR “total calcium” OR monitoring OR diagnosis OR prevention OR treatment OR “oral calcium” OR “calcium bolus” OR “dietary cation-anion difference” OR DCAD).

CAB Abstracts was searched using the same concept blocks, adapted to title, abstract, keyword, and subject heading fields where available.

The final database search was completed in February 2026. The eligible publication period was 1994–2025. This window was selected to cover the full publication-year range relevant to the current review, beginning with the earliest retained foundational source on Ca and vitamin D metabolism in dairy cows. The included scientific reports were published between 1994 and 2024.

### 2.2. Study Selection

The study selection process followed two screening stages. Titles and abstracts were screened independently by two reviewers against the eligibility criteria, followed by full-text assessment of potentially eligible reports. Disagreements were resolved through discussion and consensus. No automation tool was used to make eligibility decisions. Study authors were not contacted for additional unpublished data or missing information.

### 2.3. Inclusion and Exclusion Criteria

Eligible reports were required to meet the following criteria:

- Dairy cows (Holstein, Jersey, or crossbreeds).
- Direct relevance to clinical or subclinical hypocalcemia, including monitoring, prevention, or treatment.
- English language.
- Original diagnostic, observational, epidemiological, experimental, intervention, field, nutritional, or modeling studies for evidence synthesis; reviews and veterinary guidelines were used only for background, mechanistic interpretation, or practical context.
- Publication period 1994–2025.

Reports were excluded if they met any of the following criteria:

- Focused on other species.
- Addressed unrelated metabolic disorders.
- Lacked full-text availability.
- Were non-English reports.
- Were published before 1994 or outside the predefined eligibility role.
- Were narrative reviews, conference abstracts, or guidance documents without sufficient relevance or extractable information for the synthesis role.

### 2.4. Data Extraction and Synthesis

Data were extracted using a predefined extraction framework. Extracted items included author and year, source type, study design, country or production system when reported, animal population, parity or risk group, sample size, Ca fraction measured, timing of sampling relative to calving, diagnostic threshold, intervention or monitoring strategy, comparator or control condition when applicable, outcome measures, main findings, and limitations relevant to interpretation. Findings were organized into thematic categories: Ca homeostasis and homeorhetic adaptation, clinical and subclinical classification, monitoring indicators, risk factors, systemic effects, prevention, treatment, and practical herd-level management. Meta-analysis and formal quantitative pooling were not performed because the included reports differed substantially in design, Ca fraction measured, sampling time, diagnostic threshold, intervention or exposure definition, outcome reporting, and synthesis role. Missing or unclear information was recorded as not reported; authors were not contacted for additional data.

### 2.5. Quality Assessment and Risk of Bias

Methodological quality, risk of bias, and applicability were assessed independently by two reviewers using a structured, domain-based approach tailored to the mixed evidence base. Because the included reports comprised diagnostic and monitoring studies, observational and epidemiological studies, nutritional and treatment interventions, experimental mechanistic studies, modeling work, and review or guidance sources, no single risk-of-bias instrument was appropriate for all evidence types.

For diagnostic and monitoring studies, the appraisal domains were adapted from the Quality Assessment of Diagnostic Accuracy Studies 2 (QUADAS-2) framework and included animal or herd selection, Ca measurement as the index test, timing and frequency of sampling, definition of the diagnostic threshold or reference standard, flow and timing, completeness of reporting, and applicability to commercial dairy cow conditions. Observational and epidemiological studies were assessed for animal or herd selection, exposure and outcome measurement, control of confounding, parity or herd-level stratification, Ca-threshold definition, sampling window, completeness of reporting, and applicability.

Nutritional, intervention, experimental, and modeling studies were assessed according to group definition or allocation, intervention description, implementation or adherence monitoring, outcome measurement, follow-up, potential confounding, assumptions, completeness of reporting, and field applicability, as relevant to the study design. Narrative reviews, expert reviews, and guidance-type sources were not treated as primary evidence of intervention effectiveness. These sources were assessed for transparency, relevance, and applicability and were used only for background, mechanistic interpretation, or practical context.

Each included report was assigned an overall methodological/applicability concern rating of low, low-moderate, moderate, or high. The overall rating was determined through an integrated judgment of the severity and likely influence of limitations across the applicable appraisal domains rather than by calculating a numerical score. The rating was not used as an automatic exclusion criterion; instead, it informed the weight given to each report in the qualitative synthesis. Stronger conclusions were based on original diagnostic, epidemiological, physiological, nutritional, or intervention studies, whereas review or guidance sources and indirect evidence were used only for contextual interpretation. Disagreements between the two reviewers were resolved through discussion and consensus.

## 3. Study Selection and Evidence Base

**Figure 1:**
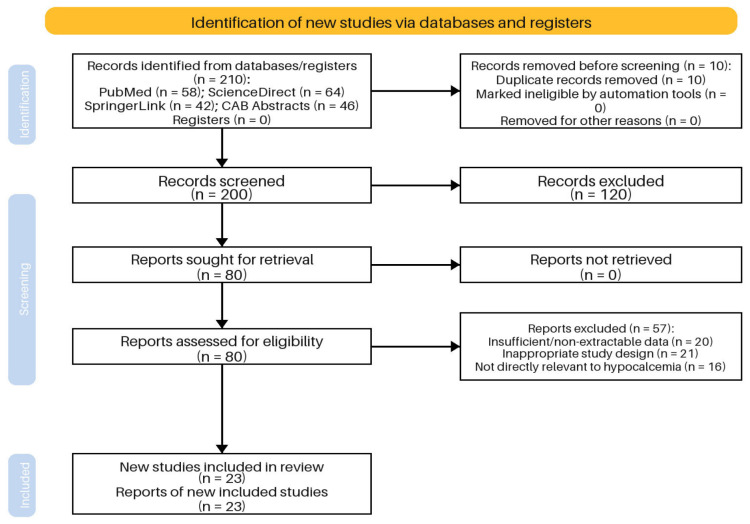
The process of identification, screening, and inclusion of publications is presented in the Preferred Reporting Items for Systematic Reviews and Meta-Analyses (PRISMA) 2020 flow diagram.

Briefly, 210 database records were identified, 200 records remained after duplicate removal, 80 full-text reports were assessed for eligibility, and 23 reports were included in the review. The complete PRISMA 2020 study-selection flow, including database-specific records and exclusion categories, is shown in Figure 1.

The 23 included reports and their report-level methodological/applicability appraisals are presented in Supplementary Table S1. Original diagnostic, epidemiological, physiological, nutritional, intervention, and field studies were used as the primary basis for qualitative synthesis. Reviews, guidelines, and extension-type sources were used only for background, mechanistic interpretation, practical context, or source identification, and their evidentiary role was explicitly considered in the methodological quality appraisal.

No quantitative synthesis or statistical meta-analysis was performed because the included evidence was heterogeneous in study design, animal population, parity structure, Ca fraction measured, diagnostic thresholds, timing of sampling, intervention protocols, and outcome definitions. Consequently, the synthesis was qualitative and claims were weighted according to evidence type, methodological concern, biological plausibility, and consistency across studies.

## 4. Monitoring and Diagnosis of Hypocalcemia

### 4.1. Physiological Background and Classification of Hypocalcemia

The evidence synthesized in this subsection was identified and selected using the search strategy and eligibility criteria described in Section 2.1, Section 2.2 and Section 2.3 and was extracted, appraised, and qualitatively analyzed according to the procedures reported in Section 2.4 and Section 2.5.

Periparturient hypocalcemia reflects the interaction between the magnitude of Ca transfer into colostrum and milk and the cow’s capacity to activate compensatory homeostatic responses. Physiological and mechanistic studies consistently identify parathyroid hormone (PTH) secretion, renal activation of vitamin D to calcitriol, renal Ca conservation, intestinal Ca absorption, and skeletal Ca mobilization as the principal mechanisms maintaining circulating Ca at the onset of lactation [1,4,5,6].

When the speed or magnitude of these responses is insufficient relative to mammary Ca export, circulating Ca declines and CHC or SCH may develop. This process is more accurately interpreted as inadequate homeorhetic adaptation to lactation than as a simple dietary mineral deficiency. Homeostasis denotes maintenance of circulating Ca within a narrow physiological range, whereas homeorhesis describes the coordinated redistribution of nutrients required to support lactation [1,4,5].

The efficiency of this adaptation is modified by mineral status, acid-base balance, and cow-level susceptibility. Magnesium (Mg) deficiency can impair both PTH secretion and tissue responsiveness to PTH, whereas a high dietary cation–anion difference (DCAD), often associated with high dietary potassium (K), can reduce PTH responsiveness through metabolic alkalosis [4,5]. Interpretation of Ca status therefore requires consideration of Mg supply, close-up ration composition, parity, breed, dry matter intake (DMI), and previous transition-cow disease [4,5].

Clinical and diagnostic studies classified hypocalcemia using a combination of clinical status and circulating Ca concentration. Clinical hypocalcemia was most commonly recognized within 24–72 h after calving and was characterized by marked reductions in tCa and iCa, accompanied by neuromuscular dysfunction [6]. Subclinical hypocalcemia was defined by reduced Ca concentrations without overt clinical signs, with commonly used early lactation thresholds of tCa ≤ 2.14 mmol/L or iCa ≤ 1.0 mmol/L [7,8,9]. Serial postpartum measurements further distinguished transient, delayed, and persistent patterns according to the timing and duration of Ca reduction [7,8].

The numerical thresholds used to classify Ca status varied substantially among studies. A practical conversion is tCa in mmol/L × 4.0 = tCa in mg/dL; thus, 2.00, 2.14, and 2.20 mmol/L correspond to approximately 8.0, 8.6, and 8.8 mg/dL, respectively. These values were applied as operational decision points rather than interchangeable biological boundaries, with selection influenced by parity, sampling time, outcome definition, and whether the objective was diagnosis, risk prediction, or herd monitoring [2,7,8,10]. tCa and iCa also differed in diagnostic interpretation. iCa is the biologically active fraction most directly associated with neuromuscular function, smooth muscle contractility, and immune cell signaling, whereas tCa is more readily measured and is therefore commonly used in herd-level investigations. However, the relationship between tCa and iCa changes around calving, and the ability of tCa to identify low iCa may be limited during the first hours and days of lactation [11,12].

Interpretation of iCa is additionally influenced by blood pH, albumin and protein binding, partial pressure of carbon dioxide (pCO_2_), bicarbonate, lactate, non-esterified fatty acids (NEFAs), sample handling, and the interval between calving and sampling [11,12]. A single low tCa value should therefore not be interpreted independently of clinical signs, parity, metabolic status, analytical conditions, and the temporal pattern of Ca reduction [7,8,11,12]. Accordingly, discordance between tCa and iCa should be interpreted as clinically meaningful information rather than as a simple analytical inconsistency, particularly in early postpartum samples in which acid–base status, protein binding, and sample handling can alter the relationship between Ca fractions [11,12].

Antepartum measurements have shown less consistent predictive value than postpartum Ca profiles. Antepartum alkaline phosphatase, tCa, and iCa did not perform as universal predictors of periparturient hypocalcemia across study populations [13]. Their use as stand-alone screening indicators is therefore limited and should be interpreted within the specific herd and sampling context.

Taken together, the available evidence supports classification based on the combined interpretation of clinical status, Ca fraction, numerical threshold, sampling time, parity, and persistence of the reduction. Variation in these factors across studies explains why no single threshold reliably distinguishes physiological adaptation from clinically relevant hypocalcemia in all cows and at all postpartum time points [2,7,8,9,10,11,12,13].

### 4.2. Clinical and Subclinical Manifestations

Clinical hypocalcemia is characterized by progressive neuromuscular dysfunction, with severity reflected by posture, muscle function, mentation, and gastrointestinal activity. The clinical continuum extends from standing cows with tremors, excitability, and gait instability to sternal recumbency with reduced muscle tone, hypothermia, tachycardia, and impaired gastrointestinal motility, and ultimately to lateral recumbency, flaccid paralysis, reduced consciousness, and death when treatment is delayed [5,6].

Stage I describes a standing but clinically abnormal cow, with signs that may include hypersensitivity, fine muscle tremors, ear twitching, mild ataxia, reduced appetite, or an unsteady gait. Stage II is characterized primarily by sternal recumbency, depression, cold extremities, tachycardia, and weak rumen contractions, whereas Stage III represents severe neuromuscular failure with lateral recumbency, flaccid paralysis, coma, or death [5,14]. Posture and neuromuscular status are therefore the principal clinical indicators of CHC severity, while biochemical values provide supportive rather than stand-alone stage definitions.

Ruminal bloat in severe CHC is consistent with impaired smooth-muscle contractility, reduced rumen motility, recumbency, and compromised eructation. Progressive gas accumulation may contribute to diaphragmatic compression and aggravate respiratory and circulatory compromise in advanced cases [5,14]. Bloat should therefore be regarded as part of the systemic neuromuscular consequences of severe hypocalcemia rather than as an unrelated digestive disorder.

Subclinical hypocalcemia, by contrast, lacks a pathognomonic clinical presentation. Reduced rumination, lower feed intake, slower gastrointestinal motility, reduced activity, and mild weakness have been reported, but these findings are subtle and non-specific [15]. Associations have also been reported between SCH and postpartum metabolic or inflammatory disorders, including ketosis, fatty liver, retained placenta, metritis, and mastitis; however, the predominantly observational evidence does not establish low Ca as an independent cause of these outcomes [6,9].

Behavioral indicators have similarly shown limited specificity. Changes in DMI, rumination, activity, or gastrointestinal motility may support clinical suspicion in high-risk cows, but they may also reflect calving stress, pain, inflammation, ketosis, lameness, or other transition period disorders [9,16]. Subclinical hypocalcemia is therefore more appropriately interpreted as a biochemical risk state than as a syndrome defined by a distinct set of observable signs.

The timing of measurement materially affects interpretation. A single sample obtained immediately after calving may capture a transient physiological nadir, whereas early sampling may fail to identify delayed or persistent SCH. Repeated postpartum measurements indicate that the significance of a low Ca value depends on its duration, day in milk, parity, and the health or production outcome under evaluation [7,8]. Time-specific or serial assessment is therefore more informative than reliance on a single universal postpartum measurement. For this reason, monitoring protocols should define whether the aim is immediate clinical detection, herd-level evaluation of a prevention program, or identification of delayed or persistent SCH before a sampling window is selected [7,8].

Table 1 summarizes the clinical stages, reported biochemical values, monitoring approaches, and immediate management implications of CHC and SCH.

Diagnostic criteria for hypocalcemia were heterogeneous across studies. Reported tCa and iCa cut-offs varied with study design, sampling time, parity structure, population characteristics, and the outcome used to define clinical relevance [7,8,9]. Consequently, prevalence estimates and risk associations are not directly comparable across all studies, and a given threshold may have different diagnostic or prognostic meaning in different postpartum contexts.

A practical approach to interpreting hypocalcemia should therefore integrate clinical status, particularly whether the cow is standing or recumbent, with the tCa or iCa concentration and the analytical conditions under which it was measured, the time relative to calving, parity and breed, Mg and acid–base status, and herd-level disease patterns. This integrated approach is more consistent with the observed heterogeneity than applying a single biochemical cut-off across all cows and sampling times. This approach is particularly important when biochemical results, clinical status, and sampling time do not align, because the same Ca concentration may have different diagnostic meaning in a standing cow sampled shortly after calving and in a high-risk cow with delayed or persistent reduction [7,8,9,10,11,12,14].

The strength of inference also varied by outcome. Most studies linking SCH with postpartum metabolic, inflammatory, and reproductive disorders were observational, which limits causal interpretation. The magnitude and consistency of reported associations differed according to threshold selection, sampling protocol, parity distribution, disease definition, and herd conditions [6,9]. These relationships should therefore be interpreted within a multifactorial transition-cow context rather than as evidence of direct causation.

Monitoring design was an additional source of heterogeneity. Single-time-point studies may classify transient reductions as clinically relevant or fail to detect later-onset SCH, whereas repeated measurements provide a more representative assessment of Ca adaptation during the first postpartum days [7,8]. This variation in study design likely contributes to differences in reported prevalence and outcome associations.

Standardized reporting of Ca fraction, analytical method, threshold, parity, and sampling time, together with greater use of repeated-measure designs, would improve comparability among future diagnostic and monitoring studies. Such standardization is necessary to distinguish transient physiological adaptation from clinically relevant delayed or persistent SCH. Without these reporting elements, differences among studies may reflect measurement timing, Ca fraction, or threshold selection rather than true biological differences in hypocalcemia risk [7,8,9,10,11,12].

## 5. Risk Factors and Monitoring of Hypocalcemia

Hypocalcemia risk reflects the interaction of cow-level susceptibility, time-dependent Ca dynamics, and herd-level nutritional management. Older and multiparous cows, Jersey cattle and their crosses, and animals with a previous history of hypocalcemia or another transition cow disease are consistently represented among higher-risk groups, while the clinical significance of these factors is modified by production level, feed intake, and the quality of close-up ration management [2,5,8,10,13,14,15,16,17].

### 5.1. Risk Factors for Hypocalcemia

Observational studies consistently associated greater susceptibility with advanced parity, Jersey breed or crossbreeding, previous hypocalcemia or transition cow disease, high milk yield, abnormal body condition score (BCS), and reduced DMI [2,8,10,13,16]. Mechanistic and management-oriented sources further identified high dietary K, inadequate Mg supply, and poorly controlled DCAD programs as factors that can impair Ca adaptation [5,14,15,17]. The relative contribution of each factor varied with parity structure, production system, timing of assessment, and the outcome used to define impaired adaptation.

Postpartum Ca measurements provided additional risk information, but their interpretation was strongly time dependent. Concentrations around 2.0–2.2 mmol/L were associated with CHC, SCH, and early-lactation health or production outcomes, although the operational threshold differed among studies according to sampling day, parity, Ca fraction, and outcome definition [7,8,10]. A single low value immediately after calving may reflect a transient physiological nadir, whereas delayed or persistent reductions can indicate a less successful adaptive response.

Nutritional and mechanistic evidence further showed that Ca adaptation is influenced by the mineral and acid–base environment before calving. High dietary K promotes metabolic alkalinity and can reduce tissue responsiveness to PTH, whereas inadequate Mg can impair both hormone secretion and target tissue responsiveness. Consequently, poorly implemented DCAD programs, inconsistent ration delivery, reduced DMI, and insufficient control of mineral composition can diminish the effectiveness of otherwise appropriate close-up diets [5,14,15,17].

Taken together, these findings support multivariable risk assessment rather than reliance on a single predictor. Cows in which parity, breed, disease history, reduced intake, and dietary risk factors cluster are appropriate candidates for closer monitoring; however, none of these variables alone provides a sufficient basis for treatment. Their clinical value depends on integration with examination findings, sampling time, herd history, and ration evaluation [7,8,14,16,18].

Herd-level investigations are most informative when Ca profiling is planned around a defined clinical question. Sampling a specified group of fresh cows at a consistent interval after calving permits comparison within parity or risk strata, whereas repeated or later sampling is required when delayed or persistent SCH is suspected [8,14]. This design reduces the interpretive error created by incidental measurements obtained at different stages of postpartum adaptation.

Urine pH has a different but complementary role. In herds using acidogenic or negative-DCAD close-up diets, it reflects the acid–base response to the ration rather than the Ca status of an individual cow. Feed and mineral analyses remain necessary because variations in K, Mg, Ca, sulfur, chloride, ration sorting, and actual DMI can alter both urine pH and the biological response to the program [14,15,17].

Interpretation of blood Ca, urine pH, and ration data should be integrated. An abnormal urine pH distribution indicates inadequate or inconsistent delivery of the intended acidogenic response, but it does not establish the presence or absence of hypocalcemia. Persistently poor fresh-cow Ca profiles, particularly when accompanied by abnormal urine pH, warrant review of dietary K, Mg, Ca and phosphorus balances, sulfur and chloride sources, palatability, sorting, bunk management, and actual feed intake [14,15,17].

### 5.2. Good Veterinary Practices for Risk-Based Management

Risk-based veterinary management distinguishes between individual-cow susceptibility and herd-level determinants of hypocalcemia. At the individual-cow level, risk is interpreted from clinical status, parity, breed, disease history, feed intake, and biochemical findings, whereas herd-level occurrence is evaluated in relation to ration composition and delivery, mineral and DCAD management, urine pH distribution, and patterns of transition-cow disease [14].

At herd level, this framework combines identification of high-risk cows with review of production and health records, reproductive performance, fresh-cow disease incidence, and ration consistency. These measures provide complementary information: clinical records indicate the expression of disease, whereas feed and management data help identify modifiable causes [14,16,18].

Across the monitoring and management literature, the most informative herd-level assessment combines CHC and SCH incidence by parity group; relapse and retreatment rates; records of metritis, mastitis, displaced abomasum, ketosis, retained fetal membranes, and culling; blood Ca profiles in defined fresh-cow groups when a problem is suspected; urine pH distributions when acidogenic diets are used; and review of close-up ration formulation and delivery [14,17,18]. Together, these indicators connect individual treatment outcomes with evaluation of the herd prevention program.

The pattern across these indicators is more informative than any single result. Increased CHC or SCH incidence despite an appropriate urine pH distribution may reflect parity structure, cow selection, concurrent disease, or the timing and interpretation of Ca sampling. Conversely, poor Ca profiles accompanied by an abnormal urine pH distribution more directly implicate ration formulation or delivery. This distinction helps prevent repeated individual Ca treatment from substituting for correction of an underlying herd-level failure [14,15,17,18].

Intervention remains dependent on clinical status. Oral Ca is most appropriately used in selected high-risk standing cows or selected SCH cases within a defined herd protocol. Recumbent or severely affected cows require prompt intravenous (IV) Ca under veterinary supervision, followed by reassessment and, when swallowing is safe, oral supplementation to reduce relapse risk [5,7,14].

Table 2 integrates these findings into a clinical decision-making framework in which risk category, clinical severity, diagnostic context, and intervention are considered together.

Table 2 emphasizes that laboratory measurements complement rather than replace clinical assessment. The same Ca concentration may have different implications according to parity, posture, neuromuscular signs, time after calving, and herd context; decisions should therefore be based on the combined pattern of risk factors and clinical findings rather than on a single biochemical value [14,18].

This distinction is particularly important for Ca supplementation. Oral Ca has the strongest practical rationale in selected high-risk cows that remain standing and can swallow safely, whereas IV Ca is reserved for clinical cases requiring rapid restoration of neuromuscular function. The latter must be administered cautiously because of the potential for cardiovascular adverse effects [5,14].

No isolated monitoring or treatment measure addresses the full problem. Clinical evaluation, selective Ca testing, targeted supplementation, ration and urine pH assessment where relevant, and analysis of herd health records provide complementary information [14,15,17,18]. An integrated, risk-based approach therefore supports both immediate case management and identification of preventable recurrence at herd level.

## 6. Systemic Effects of Hypocalcemia in Dairy Cows

Reduced Ca availability around parturition was associated with effects across digestive, metabolic, neuromuscular, reproductive, and immune systems. These effects were not uniform: their clinical relevance varied with the magnitude and duration of the Ca reduction, the timing of sampling, parity, and concurrent transition-cow stressors. Mechanistic evidence was strongest for impaired muscle and immune-cell function, whereas many reported links with postpartum disease were observational and therefore required cautious causal interpretation [5,7,8,19].

### 6.1. Digestive and Metabolic System

Experimental induction of SCH reduced DMI and rumen contractions and increased circulating NEFAs, providing direct evidence that low iCa can impair gastrointestinal motility and intensify lipid mobilization [19]. The resulting reduction in feed intake can deepen negative energy balance, increase NEFA delivery to the liver, and favor hepatic triglyceride accumulation when oxidative capacity is exceeded [19,20].

Field studies linked low periparturient Ca concentrations with metabolic outcomes, including milk loss and poorer reproductive performance, while elevated NEFAs and beta-hydroxybutyrate (BHB) were associated with displaced abomasum, clinical ketosis, metritis, and retained fetal membranes [20,21]. These findings place Ca dysregulation within a broader transition-cow metabolic network rather than support a single linear pathway from SCH to each downstream disorder. The direction and magnitude of risk are also modified by feed intake, body condition, inflammation, parity, and herd management.

### 6.2. Neuromuscular System

The clinical progression of hypocalcemia is consistent with the dependence of neurotransmitter release and excitation-contraction coupling on extracellular Ca. As iCa declines, skeletal-muscle contractility deteriorates from subtle weakness and postural instability to sternal and lateral recumbency [5,14]. The severity of posture and neuromuscular impairment is therefore more informative for clinical staging than any isolated biochemical value.

Neuromuscular effects in SCH are less specific. Reduced activity, altered rumination, mild weakness, and lower DMI may accompany low Ca status, but behavioral studies showed that these changes also occur with calving stress, pain, lameness, inflammation, ketosis, and other transition disorders [9,16]. Behavioral indicators can therefore support surveillance but cannot establish SCH without biochemical and clinical context.

### 6.3. Reproductive System

Biological plausibility for reproductive effects arises from the combined dependence of uterine contractility and postpartum immune defense on adequate Ca availability. Reduced uterine tone may delay uterine clearance, while impaired innate immune function may weaken control of bacterial contamination after calving. This dual mechanism is consistent with reported associations between low Ca status, retained fetal membranes, metritis, and delayed reproductive recovery [5,7,8,19].

Observational evidence also associated low postpartum Ca concentrations with fertility outcomes. In a large multi-herd study, low Ca concentrations during the first three postpartum weeks were associated with a lower probability of pregnancy at first artificial insemination, whereas NEFAs and BHB were not associated with that outcome in the same analysis [21]. Because these findings derive from observational data, they support prognostic relevance but do not establish a direct causal effect of low Ca on conception.

Postpartum reproductive interpretation should therefore integrate Ca status with uterine health, energy balance, inflammatory status, calving events, and herd reproductive records rather than attribute impaired fertility to Ca dysregulation alone.

### 6.4. Immune System

Experimental evidence provides the clearest mechanistic support for immune impairment. Induced SCH reduced neutrophil phagocytosis and oxidative burst after bacterial challenge, demonstrating that low iCa can directly compromise innate immune-cell function [19]. This mechanism is consistent with field associations between low Ca status and inflammatory or infectious postpartum disorders, although disease expression remains dependent on pathogen exposure, calving difficulty, hygiene, energy balance, and other co-determinants.

The timing of Ca reduction further modifies disease associations. Serial postpartum measurements indicated that Ca concentrations on later days after calving may be more informative for metritis or displaced abomasum risk than a single immediate postpartum value [7,8]. For mastitis, impaired muscle tone and immune-cell function provide biological plausibility, but teat-end condition, milking management, pathogen pressure, and overall immune status remain major determinants of clinical risk.

Taken together, the evidence supports a systemic interpretation of hypocalcemia in which low Ca contributes to reduced motility, altered energy metabolism, impaired neuromuscular function, weakened uterine clearance, and reduced innate immune competence. The strength of inference, however, differs by outcome and study design, as summarized in Table 3.

The convergence of experimental and observational findings indicates that Ca dysregulation can amplify vulnerability during early lactation, particularly when reduced feed intake, inflammation, or concurrent disease are present. The evidence is strongest for direct effects on muscle and neutrophil function; associations with ketosis, displaced abomasum, uterine disease, mastitis, and fertility are more heterogeneous and remain susceptible to confounding.

Clinical interpretation should therefore treat Ca status as one component of a wider transition-cow risk network. Early detection is relevant when it identifies delayed or persistent Ca dysregulation in cows with compatible clinical, metabolic, or herd-level findings, rather than when a single low value is considered in isolation.

### 6.5. Integrated Interpretation and Evidence Limitations

Subclinical hypocalcemia was reported more frequently than CHC, particularly in multiparous cows, but its biological significance varied with sampling time, parity, analytical threshold, Ca fraction, and persistence pattern [2,7,8,11,12]. A transient early postpartum decline may represent short-lived adaptation, whereas delayed or persistent reductions are more consistently associated with adverse health outcomes.

Associations with ketosis, displaced abomasum, metritis, retained fetal membranes, mastitis, and reproductive dysfunction were not uniform across studies. Differences in study design, sampling schedules, parity distribution, disease definitions, analytical thresholds, and herd conditions limited direct comparison and weakened causal inference [7,8,20,21]. Accordingly, these outcomes should be interpreted as multifactorial disorders in which Ca dysregulation may contribute without acting as an exclusive cause.

Diagnostic and prognostic performance therefore depends on measurement strategy: serial or time-specific assessment better captures the duration and trajectory of Ca adaptation than a single immediate postpartum sample, while behavioral changes remain supportive but non-specific alerts [7,8,9,16].

The combined evidence favors risk-based interpretation over universal threshold-driven intervention. Clinical status, parity, Ca fraction, sampling time, feed intake, uterine health, immune function, and herd disease patterns should be considered together when deciding whether low Ca represents physiological adaptation or clinically relevant dysfunction.

The principal evidence limitation was methodological heterogeneity across thresholds, sampling days, Ca fractions, disease outcomes, and study populations. Further controlled and commercial-field studies should use standardized definitions and repeated Ca profiling to distinguish transient from delayed or persistent SCH and to determine whether integrated prevention strategies improve health and production outcomes.

## 7. Preventive and Therapeutic Strategies for Hypocalcemia

Evidence from nutritional, physiological, intervention, and management-oriented reports indicates that prevention is most effective when prepartum and early-postpartum measures are coordinated rather than applied as isolated interventions. Controlled prepartum DCAD, adequate Mg supply, avoidance of excessive dietary K, maintenance of DMI, and consistent clinical and biochemical monitoring form the principal components of this approach because each addresses a different determinant of Ca adaptation around calving [5,10,14,15,17].

The nutritional evidence consistently links the effectiveness of negative-DCAD programs to the degree of metabolic acidification actually achieved, rather than to the formulated DCAD value alone. Group-level urine pH and ration analysis are therefore needed to interpret program performance, particularly in relation to K, sulfur, chloride, Mg, Ca, and phosphorus. Inadequate Mg and excessive dietary K can impair PTH secretion or tissue responsiveness and thereby weaken Ca mobilization, whereas excessive acidification or reduced ration palatability may compromise DMI and offset the intended benefit. Vitamin D pathways remain physiologically relevant to intestinal Ca absorption, but the available evidence supports their use only within carefully timed and controlled protocols rather than as an unrestricted primary preventive measure [4,5,6,14,15,17].

The intervention and economic evidence does not support a uniform supplementation strategy for all fresh cows. Oral Ca is most defensible in standing, non-recumbent cows with identifiable risk factors around calving, and as follow-up after treatment of clinical cases when swallowing is safe. Economic modeling and randomized evidence indicate that blanket supplementation of clinically normal cows may provide limited or variable benefit, whereas risk-targeted protocols are more likely to concentrate treatment in animals expected to benefit [3,15,22]. By contrast, IV Ca remains the treatment of choice for recumbent cows or those with severe clinical weakness because it rapidly restores circulating Ca; however, the need for skilled administration, cardiovascular monitoring, relapse prevention, and correction of the underlying herd-level problem limits its role to clinical treatment rather than routine prevention [5,14].

Postpartum monitoring is most informative when it evaluates whether the prepartum program has translated into adequate Ca adaptation in defined risk groups. Close-up ration review, urine pH and DMI assessment after adaptation to an acidogenic diet, clinical examination at calving, and selective Ca testing during the first 24–72 h postpartum provide complementary information. Repeated or later testing is particularly relevant when SCH is suspected to persist or to become evident after the immediate postpartum period. Rumination and activity data may support case detection, but their limited specificity means that they should complement, rather than replace, clinical assessment and biochemical interpretation [7,8,9,12,13,16,18].

Table 4 integrates the evidence according to timing and clinical status. Prepartum nutritional measures operate primarily at herd level, whereas oral and IV Ca are individual-cow interventions whose use depends on risk classification and disease severity. The combined evidence therefore favors protocols that link ration implementation, monitoring of biological response, selective supplementation, and prompt treatment of clinical cases, rather than reliance on a single product or a single postpartum Ca measurement.

The strongest and most consistent support is for controlled prepartum nutritional management, particularly DCAD programs verified by urine pH and ration monitoring, and for prompt IV Ca treatment in true clinical cases. The effect of oral Ca is more heterogeneous and depends on parity, previous disease history, production level, lameness or reduced intake, and timing relative to calving. Evidence for supportive postpartum interventions directed at inflammation or general transition health is less specific to Ca homeostasis; such measures should therefore not be interpreted as hypocalcemia prevention unless Ca-related outcomes are directly assessed. Overall, the findings support a risk-based, time-dependent strategy in which preventive nutrition, monitoring, and treatment are matched to the biological and clinical context of the cow [3,5,7,8,10,14,15,17,22,23].

## 8. Conclusions

This systematic review indicates that hypocalcemia in high-producing dairy cows should be interpreted as a disorder of transition-cow adaptation rather than only as a reduction in blood Ca concentration. Clinical hypocalcemia remains an emergency condition, whereas SCH is more frequent and more difficult to interpret because its biological relevance depends on parity, sampling time, Ca fraction, threshold used, and whether the reduction is transient, delayed, or persistent.

The mechanistic evidence supports a biologically coherent link between impaired Ca availability and reduced smooth-muscle function, lower DMI, altered energy metabolism, immune-cell dysfunction, uterine disease risk, and poorer reproductive outcomes. However, many field associations are modified by diet, parity, BCS, calving events, inflammation, herd management, and diagnostic timing. Therefore, the strongest conclusion is not that low Ca alone explains postpartum disease, but that inadequate Ca adaptation is an important component of a broader transition-cow risk network.

From a management perspective, the most defensible strategy is an integrated, risk-based protocol that combines close-up ration control, DCAD and Mg management, urine pH and feed monitoring, selective postpartum Ca assessment, oral Ca for appropriate high-risk standing cows, and IV Ca for true clinical cases. Because the included studies differed in thresholds, sampling schedules, Ca fractions, outcomes, and intervention protocols, the evidence was not suitable for a robust quantitative synthesis. Future research should prioritize standardized diagnostic definitions, repeated Ca profiling, distinction between transient and persistent SCH, and controlled field evaluations of combined prevention programs.

## Figures and Tables

**Table 1:** Classification and clinical characteristics of hypocalcemia in dairy cows.

Category	Reported tCa (mmol/L; mg/dL)	Reported iCa (mmol/L)	Key Clinical Signs	Monitoring Approach	Immediate Management Implication
CHC—Stage I	≤2.0; ≤8.0	≤1.0	Standing cow; tremors, excitability, ataxia, unsteady gait	Clinical observation; tCa/iCa if available	Oral Ca if swallowing is safe; close monitoring [5,6,14]
CHC—Stage II	≤1.8; ≤7.2	≤0.8	Sternal recumbency, cold extremities, weak rumen motility, tachycardia	Clinical exam; Ca confirmation if it does not delay treatment	Veterinary Ca therapy; intravenous (IV) Ca when indicated + oral follow-up [5,14]
CHC—Stage III	≤1.5; ≤6.0	≤0.6	Lateral recumbency, flaccid paralysis, severe depression/coma	Emergency clinical diagnosis and monitoring	Emergency IV Ca under veterinary supervision [5,14]
SCH	Common operational cut-offs: 2.0–2.14; 8.0–8.6	Often <1.0; pH-sensitive	No overt signs; possible reduced DMI, rumination, activity, or motility	Selective/serial tCa or iCa in high-risk cows or problem herds	Risk-based oral Ca, dietary review, and herd monitoring [7,8,9,10,11,12,15]

Note: CHC staging is primarily clinical; the reported Ca concentrations are approximate and should not be interpreted as universally validated stage-specific diagnostic cut-offs. Thresholds for SCH are operational and vary with parity, sampling time, analytical method, study population, and outcome definition [5,7,8,9,14]. Abbreviations: calcium (Ca); clinical hypocalcemia (CHC); dry matter intake (DMI); ionized calcium (iCa); intravenous (IV); subclinical hypocalcemia (SCH); total calcium (tCa).

**Table 2:** Clinical Decision-Making Framework for Risk-Based Management of Hypocalcemia.

Clinical Status/Risk Category	Key Indicators	Diagnostic Approach	Intervention Strategy	Clinical Rationale
**Low-risk fresh cows**	Normal appetite and activity; no major parity, breed, disease history, or ration risk	Routine observation and fresh-cow checks	No routine Ca supplementation	Avoid unnecessary treatment when physiological adaptation is adequate [3,14,15]
**High-risk cows**	Multiparous/older, Jersey/crossbred, previous CHC/SCH, high yield, abnormal BCS, low DMI, high-K or poorly controlled DCAD diet	Risk record review; clinical exam; selective tCa/iCa testing; urine pH if negative DCAD diet is used	Optimize close-up ration, Mg and DCAD control; oral Ca at calving/early postpartum when included in herd protocol	Targets cows most likely to benefit while preserving herd-level prevention [2,8,10,13,14,15,16,17]
**Suspected SCH**	No overt signs; reduced DMI, rumination, activity, or slow motility in a high-risk cow	Selective or serial tCa/iCa testing interpreted by sampling time, parity, pH and herd context	Risk-based oral Ca, close monitoring, and ration/disease record review	Reduces misclassification from a single time-point measurement [7,8,9,11,12,16]
**CHC—mild/moderate**	Standing weakness or sternal recumbency, cold extremities, weak rumen motility	Clinical exam; Ca confirmation if it does not delay treatment	Veterinary-directed Ca therapy; oral follow-up if safe	Early correction may prevent deterioration and relapse [5,14]
**CHC—severe**	Lateral recumbency, flaccid paralysis, severe depression or coma	Immediate clinical diagnosis and monitoring	IV Ca under veterinary supervision plus supportive care	Rapid restoration of neuromuscular function is required [5,14]
**Post-treatment management**	Recovery after CHC; relapse risk; reduced intake or persistent weakness	Clinical reassessment; repeat Ca testing if response is incomplete or relapse is suspected	Oral Ca follow-up, feed/water access, and monitoring for secondary disease	Stabilizes Ca status and detects persistent adaptation failure [5,14]
**Herd-level management**	High CHC/SCH incidence; metritis, mastitis, ketosis, displaced abomasum, retained placenta, or poor fresh-cow performance	Blood Ca profile in defined groups; urine pH distribution; feed/mineral analysis; health record audit	Revise DCAD, Mg, dietary K, ration delivery, cow comfort, standard operating procedures (SOPs) and supplementation criteria	Connects individual cases to preventable herd-level causes [14,17,18]

Note: Abbreviations: body condition score (BCS); calcium (Ca); clinical hypocalcemia (CHC); dietary cation–anion difference (DCAD); dry matter intake (DMI); ionized calcium (iCa); intravenous (IV); magnesium (Mg); potassium (K); standard operating procedures (SOPs); subclinical hypocalcemia (SCH); total calcium (tCa).

**Table 3:** Systemic Effects of Hypocalcemia and Clinical Implications.

Physiological System	Key Mechanism	Clinical Consequences	Veterinary Implications	Evidence Interpretation
Digestive and metabolic	Reduced iCa impairs smooth-muscle motility, lowers DMI, and increases lipid mobilization	Reduced intake, deeper negative energy balance, and reported associations with ketosis, fatty liver, and displaced abomasum	Interpret Ca with DMI, rumination, rumen fill, and BHB/NEFAs when clinically indicated	Experimental evidence supports effects on motility and intake; downstream disease associations are multifactorial [19,20,21]
Neuromuscular	Impaired neurotransmitter release and excitation–contraction coupling	Weakness, reduced activity, postural instability, and progression to sternal or lateral recumbency	Base clinical staging primarily on posture and neuromuscular status; treat severe cases promptly	Clinical signs support CHC severity assessment; behavioral changes alone are not specific for SCH [5,14,19]
Reproductive	Reduced uterine tone combined with impaired innate immune defense	Reported associations with retained fetal membranes, delayed uterine clearance, metritis, and poorer fertility indicators	Interpret Ca together with uterine health, energy balance, calving events, and reproductive records	Evidence is mainly associative; causal inference is limited by multiple postpartum co-determinants [7,8,19,21]
Immune	Reduced neutrophil phagocytosis and oxidative burst	Biological plausibility for greater susceptibility to metritis, mastitis, and persistent postpartum inflammation	Combine Ca assessment with disease records, hygiene, pathogen exposure, and herd-level prevention	Experimental evidence supports immune impairment; field disease risk remains multifactorial [7,8,19]

Note: Abbreviations: beta-hydroxybutyrate (BHB); clinical hypocalcemia (CHC); dry matter intake (DMI); ionized calcium (iCa); non-esterified fatty acids (NEFAs); subclinical hypocalcemia (SCH).

**Table 4:** Risk-Based Timeline for Prevention, Monitoring, and Treatment of Hypocalcemia.

Timing/Trigger	Main Objective	Action/Monitoring	Target Cows/Group for This Timing/Trigger	Evidence-Based Caution for This Timing/Trigger
Close-up period (last 2–3 wk before expected calving)	Prepare Ca homeostasis before colostrogenesis and lactation demand	Formulate controlled DCAD ration; avoid excess K; ensure Mg adequacy; protect DMI and palatability	Target group for close-up period: All close-up cows, especially multiparous cows and high-risk herds	Caution for close-up period: Success depends on actual ration minerals and implementation, not the declared DCAD value alone [4,5,6,14,15,17]
Late close-up verification	Confirm acid-base response and feed intake	Evaluate urine pH distribution, feed sorting, refusals, BCS, and close-up pen comfort	Target group for late close-up verification: Close-up group managed on acidogenic diet	Caution for late close-up verification: Over-acidification or poor palatability can reduce DMI and undermine prevention [14,15,17]
Calving to first 24 h postpartum	Identify clinical cases and support selected high-risk cows	Clinical examination; oral Ca according to SOPs; blood Ca when indicated	Target group for calving to first 24 h postpartum: Multiparous cows, previous CHC/SCH, Jersey-type cows, high yield, lameness, low intake, difficult calving	Caution for calving to first 24 h postpartum: Blanket supplementation has variable value; benefit is most defensible when risk-targeted [3,5,7,8,14,15]
24–72 h postpartum	Detect delayed or persistent SCH and metabolic coupling	Selective tCa/iCa testing; interpret with DMI, rumination, BHB/NEFAs, and disease records	Target group for 24–72 h postpartum: Cows with poor intake, disease signs, high-risk status, or herd-level concern	Caution for 24–72 h postpartum: Single time-point testing can misclassify cows; interpret with parity and sampling time [7,8,9,11,12,16,18]
CHC	Rapidly restore circulating Ca and prevent relapse	Slow IV Ca by trained personnel; follow with oral Ca/supportive care when safe	Target group for CHC trigger: Recumbent or severely weak cows	Caution for CHC trigger: IV Ca is emergency treatment, not routine prevention; monitor cardiovascular risk [5,14]
Herd follow-up	Prevent recurrence at system level	Audit CHC/SCH incidence, urine pH, ration K/Mg/DCAD, calving pen management, and oral Ca SOPs	Target group for herd follow-up: Herds with clustered cases or unsatisfactory transition outcomes	Caution for herd follow-up: Repeated rescue treatment signals prevention failure and requires ration/management correction [14,17,18]

Note: Abbreviations: beta-hydroxybutyrate (BHB); body condition score (BCS); clinical hypocalcemia (CHC); dietary cation–anion difference (DCAD); dry matter intake (DMI); ionized calcium (iCa); intravenous (IV); magnesium (Mg); non-esterified fatty acids (NEFAs); potassium (K); standard operating procedures (SOPs); subclinical hypocalcemia (SCH); total calcium (tCa).

## Data Availability

No new primary data were generated in this study.

## Supplementary Materials

The following supporting information can be downloaded at: https://www.mdpi.com/article/10.3390/life16071082/s1. Reference [24] is cited in the Supplementary Materials.
