# Supplementary material for: Hypocalcemia in Dairy Cows: A Systematic Review of Metabolic Implications and Management Strategies
Source: Life (Basel). 2026 Jun 28;16(7):1082. doi: 10.3390/life16071082 (PMC13412600; doi:10.3390/life16071082)
Supplement: Supplementary file 1 [file life-16-01082-s001.zip › life-4413865-supplementary.pdf]

Supplementary Table S1. Quality assessment of included reports using a simplified QUADAS-2-adapted and domain-based framework

Included report	Selection / applicability	Index test, exposure, or intervention	Reference standard / outcome definition	Flow, timing, and reporting	Overall concern
[1] Goff and Horst 1997	N/A	N/A	N/A	N/A	Moderate
[2] Reinhardt et al. 2011	Moderate	Low	Moderate	Moderate	Moderate
[3] McArt and Oetzel 2015	N/A	Moderate	Moderate	Moderate	Moderate
[4] Hernandez Castellano et al. 2020	N/A	N/A	N/A	N/A	Moderate
[5] Goff 2014	N/A	N/A	N/A	N/A	Moderate
[6] Horst et al. 1994	N/A	N/A	N/A	N/A	Moderate
[7] Neves et al. 2018	Low-moderate	Low	Low-moderate	Low-moderate	Low-moderate
[8] Neves et al. 2018	Low-moderate	Low	Low-moderate	Low-moderate	Low-moderate
[9] Hendriks et al. 2020	Low-moderate	Moderate	Moderate	Moderate	Moderate
[10] Melendez et al. 2022	Moderate	Low	Moderate	Moderate	Moderate
[11] Ott et al. 2021	Low-moderate	Low	Low-moderate	Low-moderate	Low-moderate
[12] Cohrs et al. 2023	Low-moderate	Low	Low-moderate	Low-moderate	Low-moderate
[13] Arnold et al. 2024	Low-moderate	Low	Moderate	Low-moderate	Moderate
[14] Goff 2008	N/A	N/A	N/A	N/A	Moderate
[15] Vieira-Neto et al. 2024	N/A	N/A	N/A	N/A	Moderate
[16] Emam et al. 2023	Low-moderate	Moderate	Moderate	Low-moderate	Moderate
[17] Zhang et al. 2022	Low	Low	Low-moderate	Low-moderate	Low-moderate
[18] LeBlanc 2010	N/A	N/A	N/A	N/A	Moderate
[19] Martinez et al. 2014	Low-moderate	Low	Low	Low-moderate	Low-moderate
[20] Ospina et al. 2010	Moderate	Low	Moderate	Moderate	Moderate
[21] Chapinal et al. 2012	Moderate	Low	Moderate	Moderate	Moderate
[22] Couto Serrenho et al. 2023	Low-moderate	Low	Low-moderate	Low-moderate	Low-moderate
[23] Barragan et al. 2020	Low-moderate	Low	Low-moderate	Low-moderate	Moderate

Legend: Low = low methodological or applicability concern; Low-moderate = minor limitations unlikely to change interpretation; Moderate = notable design, reporting, applicability, or indirectness limitations requiring cautious interpretation; High = major concern; N/A = domain not applicable to the evidence type. Review, guidance, and modeling reports were not treated as primary diagnostic or intervention-effectiveness studies; N/A therefore reflects design applicability, not exclusion. The MDPI Publisher's Note is not a scientific report and was not appraised.

Assessment approach: QUADAS-2 domains were adapted for diagnostic and monitoring reports. For observational, experimental, intervention, modeling, review, and guidance-type reports, the same domain structure was applied as a structured methodological-quality and applicability appraisal.
